# Effects of exercise-induced fatigue and lunge direction on racket-side lower-limb biomechanics during forward lunges in male badminton athletes

**DOI:** 10.3389/fbioe.2026.1967879

**Published:** 2026-10-27

**Authors:** Zhonghao Xie, Ziyang Feng, Yang Qiu, Zijian Jin, Wei Li, Shikun Xu, Jieyi Zhang, Xiang Huang, Yan Wang, Zhiguan Huang

**Affiliations:** 1 Guangdong Provincial Engineering Technology Research Center for Sports Assistive Device, Guangzhou Sport University, Guangzhou, China; 2 School of Physical Education, Guangzhou Sport University, Guangzhou, China; 3 Department of Sports Science, Hanyang University, Seoul, Republic of Korea; 4 National Badminton Academy, Guangzhou Sport University, Guangzhou, China; 5 School of Sport and Health, Guangzhou Sport University, Guangzhou, China; 6 Department of Biomedical Engineering and Sports Technology, The Hong Kong Polytechnic University, Hong Kong SAR, China

**Keywords:** badminton, center-of-mass, dynamic postural stability, fatigue, lunge direction

## Abstract

**Purpose:**

This study examined the effects of exercise-induced fatigue and lunge direction on racket-side lower-limb biomechanics during forehand and backhand forward lunges in male badminton athletes.

**Methods:**

Twenty-four male athletes performed standardized three-step lunges before and after a combined sprint-interval running and maximal vertical-jump fatigue protocol. Three-dimensional kinematics were recorded using a Vicon motion-capture system, while ground reaction forces were recorded using force platforms. Lower-limb biomechanical analyses focused on the racket-side limb. Outcome variables included phase durations, joint kinematics and moments, center-of-mass (COM) velocity, ground reaction forces (GRFs), impulses, joint quasi-stiffness, and dynamic postural stability. Outcomes were analyzed using two-way repeated-measures ANOVA with prespecified biomechanical-domain- and effect-specific Benjamini–Hochberg false discovery rate correction.

**Results:**

No statistically significant fatigue × direction interactions were detected after BH correction; however, given the limited statistical sensitivity for interaction effects, these findings should be considered inconclusive. Fatigue shortened braking duration, reduced recovery-phase COM velocity gain, braking-phase impulses, recovery-phase anteroposterior impulse, and selected joint ROMs, while increasing recovery-phase hip and knee quasi-stiffness. Backhand lunges showed greater secondary-impact GRF, higher DPSI, VSI and MLSI values, greater ankle dorsiflexion and inversion, and a greater knee extension moment than forehand lunges.

**Conclusion:**

Within the racket-side limb, fatigue induced a more constrained lunge strategy and reduced recovery-phase impulse generation and COM velocity increase. Backhand lunges showed greater impact loading, braking-phase force fluctuations, and ankle mechanical demands than forehand lunges.

## 1 Introduction

Badminton involves repeated high-intensity, multidirectional movements, with lunges accounting for more than 15% of movements during match play (Kuntze et al., 2010; Phomsoupha and Laffaye, 2015). The lunge stance phase comprises braking and recovery phases. During braking, players decelerate the body and absorb impact before stroke execution, whereas during recovery, they generate force to regain velocity for subsequent repositioning (Lam et al., 2016; Xie et al., 2025c). Ground reaction forces may reach approximately 2.5 times body weight during lunging, requiring substantial eccentric control during weight acceptance (Abián-Vicén et al., 2012; Bravo-Sánchez et al., 2020). Previous studies have further demonstrated that badminton lunges involve substantial knee and ankle joint demands, rapid deceleration, and effective control of whole-body momentum during the braking and recovery phases of directional movements (Kuntze et al., 2010; Maloney, 2018). These characteristics highlight the importance of maintaining effective lower-limb movement strategies during badminton-specific tasks.

Lunge direction may influence lower-limb loading and movement strategy. Badminton lunges are commonly classified into six directions, with forehand and backhand forward lunges performed on the racket and non-racket sides, respectively (Lee and Loh, 2019). These movements generate substantial plantar loading and may impose different joint-loading and stability requirements because of their different orientations relative to the racket side (Hong et al., 2014; Hu et al., 2015; Lee and Loh, 2019). However, previous findings are inconsistent. Nielsen et al. (2020) reported lower frontal-plane hip, knee, and ankle moments during backhand than forehand lunges in male badminton players, whereas Xie et al. (2025a) found no directional differences in frontal-plane joint moments among female amateur players. These discrepancies may reflect differences in participant sex, skill level, movement execution, or experimental procedures. Therefore, these inconsistent findings highlight the need to further clarify direction-specific biomechanical demands during badminton lunges. However, evidence regarding direction-specific differences in trained male badminton athletes remains limited.

Fatigue may further alter lunge biomechanics. Lower-limb injuries account for approximately 52% of badminton-related injuries, and fatigue may contribute to altered loading and movement control (Valldecabres et al., 2022; Marchena-Rodriguez et al., 2024). Repeated lunges performed to fatigue shift plantar pressure toward the medial midfoot of the trail limb, suggesting altered load distribution and postural control (Valldecabres et al., 2022). Isolated ankle-dorsiflexor fatigue has also been shown to impair forehand-lunge performance and increase impact forces during backhand lunges (Tong et al., 2023). However, fatigue responses depend on task intensity and duration, the muscles involved, and the movement conditions under which fatigue is induced (Paillard, 2012; Roth et al., 2021). The fatigue protocol must therefore be considered when interpreting biomechanical changes.

Badminton match play consists of short-duration, high-intensity actions involving repeated running, jumping, and rapid changes of direction (Cabello Manrique and González-Badillo, 2003; Phomsoupha and Laffaye, 2015; Valldecabres et al., 2022). Accordingly, a fatigue protocol combining sprint-interval running and maximal vertical jumps may reproduce selected lower-limb fatigue characteristics relevant to badminton under controlled laboratory conditions (Chappell et al., 2005; Tsai et al., 2009). Using this protocol, Xie et al. (2025a) reported reduced knee flexion and increased leg stiffness in amateur badminton players. However, biomechanical characteristics during badminton lunges may vary with competitive level (Lam et al., 2020), and it remains unclear whether the fatigue-related responses reported in amateur players are also present in highly trained male athletes. Moreover, the previous study focused primarily on conventional joint-level biomechanical variables and did not characterize phase-specific changes in whole-body COM velocity, braking and recovery impulses, or dynamic postural stability. These variables may provide additional insight into the mechanical transition from impact attenuation to movement recovery during a badminton lunge. Examining the independent and interactive effects of fatigue and direction may help clarify direction-specific loading, stability-control strategies, and fatigue-management approaches in badminton.

Therefore, this study examined racket-side lower-limb kinematics and kinetics during forehand and backhand forward lunges performed by trained male badminton athletes under non-fatigued and fatigued conditions. We hypothesized that fatigue would alter racket-side lower-limb movement strategies and modify lower-limb mechanical demands. We further hypothesized that backhand lunges would impose greater racket-side loading and stability-control demands than forehand lunges.

## 2 Methods

### 2.1 Participants

An *a priori* sample-size calculation was performed using G*Power 3.1. In the absence of a reliable prior effect-size estimate, a conventional medium effect size (f = 0.25) was assumed, with α = 0.05, power = 0.80, a correlation of 0.50 among repeated measures, and a nonsphericity correction of 1.0. The original calculation treated the four experimental conditions as repeated measurements and yielded a minimum sample size of 24 participants. Accordingly, 24 male badminton athletes from Guangzhou Sport University participated in the study (age: 22.83 ± 3.21 years; body mass: 70.46 ± 7.90 kg; height: 1.76 ± 0.06 m; badminton experience: 11.58 ± 3.92 years).

Participants were eligible if they held a Chinese National Second-Class Athlete certification or higher, completed at least 6 h of badminton-specific training per week, were right-handed, and had sustained no lower-limb injury during the preceding 6 months. According to the Participant Classification Framework (McKay et al., 2022), they were classified as Tier three athletes (highly trained).

The study was approved by the Ethics Committee of Guangzhou Sport University (approval number: 2026LCLL-111) and conducted in accordance with the Declaration of Helsinki. All participants provided written informed consent before participation.

### 2.2 Experimental setup and protocol

Participants were instructed to avoid strenuous physical activity for at least 24 h before testing. On arrival, they were fitted with Suunto heart-rate monitors (Suunto Oy, Vantaa, Finland) for continuous monitoring and completed a standardized 15-min warm-up comprising dynamic stretching and 20 practice lunges.

Participants performed standardized three-step forehand and backhand forward lunges (Hong et al., 2014). Lunge distance was set at 1.5 times individual leg length, defined as the vertical distance from the anterior superior iliac spine to the ground (Kuntze et al., 2010; Lee and Loh, 2019). All participants wore appropriately sized Yonex badminton shoes of the same model (SHB101CR). Shuttlecocks were delivered using an ADIBO badminton shuttlecock machine (A210) positioned at a fixed location and configured to target standardized forehand and backhand front-court areas at consistent speed, trajectory, and frequency (Figure 1).

**FIGURE 1:**
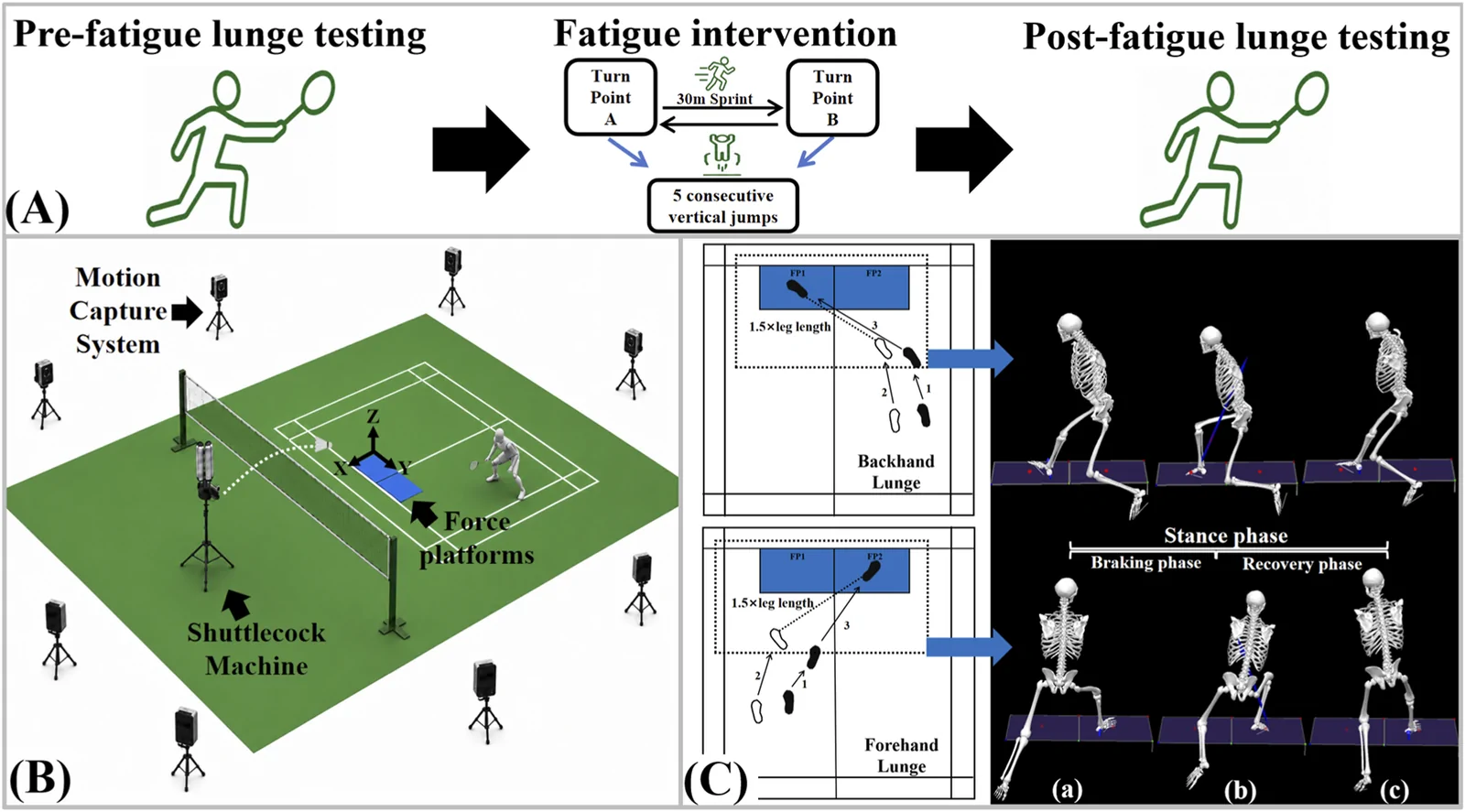
Experimental procedure, setup, and forward lunge task. **(A)** Testing procedure, including pre-fatigue lunge testing, fatigue intervention, and post-fatigue lunge testing. **(B)** Laboratory setup, including the motion capture system, force platforms, shuttlecock machine, and global coordinate system. **(C)** Schematic illustration of the forehand and backhand forward lunge tasks. White and black footprints indicate left and right foot positions, respectively, and numbers indicate the step sequence. (a) Initial contact, (b) peak knee flexion, and (c) toe-off. The intervals a–b, b–c, and a–c represent the braking, recovery, and stance phases, respectively.

Forty-three reflective markers (14 mm in diameter) were attached to anatomical landmarks to define the body segments and joint coordinate systems (Xie et al., 2025a). Three-dimensional marker trajectories were recorded at 200 Hz using an eight-camera Vicon motion-capture system (Oxford Metrics Ltd., Oxford, UK), while ground reaction forces were recorded at 1000 Hz using two AMTI force platforms (AMTI, Watertown, MA, USA) embedded flush with a simulated badminton court. Marker trajectories and ground reaction force data from the force platforms were simultaneously recorded within the Nexus acquisition environment, ensuring temporal alignment based on the same acquisition clock. In the global coordinate system, the x-axis represented the anteroposterior direction, with positive x directed toward the net; the y-axis represented the mediolateral direction, with positive y directed to the participant’s left when facing the net; and the z-axis represented the vertical direction, with positive z directed upward (Figure 1).

During pre-fatigue testing, participants started from a standardized position, performed a three-step forward lunge, landed with the racket-side foot on the center of the force platform, struck the shuttlecock, and returned to the starting position as quickly as possible (Lam et al., 2016). Within each testing condition, the six lunge trials (three forehand and three backhand) were individually randomized using an online randomization tool (www.random.org). Separate randomization sequences were generated for the pre-fatigue and post-fatigue sessions. A 30-s rest interval was provided between pre-fatigue trials. A trial was considered valid when the participant: (1) started with the foot correctly positioned at the designated line; (2) landed the racket-side foot fully within the force-platform target area; (3) successfully struck the shuttlecock; and (4) returned to the starting position without an additional corrective step. Trials were considered invalid if any of these criteria were not met or if obvious foot slippage or a clear discontinuity in the movement sequence was observed. Invalid trials were discarded and repeated until three valid trials were obtained for each lunge direction in each testing condition.

### 2.3 Fatigue protocol

Exercise-induced fatigue was elicited using repeated vertical jumps and 30-m sprints (Chappell et al., 2005; Xie et al., 2025a). Maximum vertical-jump height was defined as the greatest height achieved across three maximal countermovement jumps. Each fatigue cycle comprised five consecutive vertical jumps followed immediately by a maximal-effort 30 m sprint. At the end of each fatigue cycle, jump height, heart rate, and RPE were assessed, and the protocol continued until all predefined fatigue criteria were met: (1) jump height below 70% of maximum for five consecutive jumps; (2) RPE ≥17 on the Borg 6–20 scale; and (3) heart rate ≥80% of estimated maximum heart rate, calculated as 220 − age.

Post-fatigue testing was initiated immediately after the fatigue criteria were met. Because the fatigue intervention was conducted in the corridor immediately outside the laboratory, participants returned directly to the testing position and began the first post-fatigue lunge within approximately 10 s. Participants then completed three valid forehand and three valid backhand trials according to the randomized post-fatigue order, with no scheduled rest between trials. Heart rate and RPE were assessed immediately before each post-fatigue trial. Invalid trials were repeated according to the predefined validity criteria.

### 2.4 Data reduction

Marker trajectories were manually labeled in Vicon Nexus (Oxford Metrics Ltd., Oxford, UK) and exported to Visual3D (C-Motion Inc., Rockville, MD, USA). Kinematic and kinetic data were low-pass filtered using fourth-order Butterworth filters with cutoff frequencies of 12 and 100 Hz, respectively, consistent with previous badminton-lunge research using the same cutoffs (Lam et al., 2016) and the frequency-based approach of Nigg et al. (2009).

The stance phase was divided into braking and recovery phases (Lee and Loh, 2019). Initial contact (IC) was defined as the first frame in which vertical ground reaction force (GRF) exceeded 15 N, peak knee flexion (PKF) as the frame of maximum racket-side knee flexion, and toe-off (TO) as the first frame in which vertical GRF fell below 15 N. The braking, recovery, and stance phases were defined as IC–PKF, PKF–TO, and IC–TO, respectively (Figure 1).

Although both limbs were instrumented with reflective markers as part of the motion-capture model, the present lower-limb biomechanical analyses focused exclusively on the racket-side limb; the contralateral trail limb was not included in the outcome analyses. Outcome variables included phase durations, anteroposterior center-of-mass (COM) velocity, peak joint angles, joint range of motion (ROM), GRFs and impulses, peak net internal joint moments, phase-specific sagittal-plane joint quasi-stiffness, and the anteroposterior (APSI), mediolateral (MLSI), vertical (VSI), and dynamic postural stability indices (DPSI) (Equations 1–6).

$$J_{dir}=\int_{t1}^{t2}F_{dir}\left(t\right)dt$$ (1)

where J_dir_ is the impulse in the specified direction, F_dir(t)_ is the normalized GRF, and t1 and t2 denote the start and end of the analyzed phase, respectively.

$$K_{\text{QS}}=\frac{△M}{△\theta }$$ (2)

K_QS_, joint quasi-stiffness; ΔM, change in joint moment, ΔM = M_max_ - M_min_; Δθ, change in joint angle, Δθ = θ_max_ - θ_min_. This calculation was performed separately for the braking phase and recovery phase.

$$\text{APSI}=\sqrt{\frac{\sum {\left(0-GRFx\right)}^{2}}{\begin{array}{cc} \text{Number} & \begin{array}{ccc} \text{of} & \text{data} & \text{points} \end{array} \end{array}}}÷\begin{array}{cc} \text{Body} & \text{Weight} \end{array}$$ (3)

$$\text{MLSI}=\sqrt{\frac{\sum {\left(0-GRFy\right)}^{2}}{\begin{array}{cc} \text{Number} & \begin{array}{ccc} \text{of} & \text{data} & \text{points} \end{array} \end{array}}}÷\begin{array}{cc} \text{Body} & \text{Weight} \end{array}$$ (4)

$$\text{VSI}=\sqrt{\frac{\sum {\left(\text{BW}-\text{GRFz}\right)}^{2}}{\begin{array}{cc} \text{Number} & \begin{array}{ccc} \text{of} & \text{data} & \text{points} \end{array} \end{array}}}÷\begin{array}{cc} \text{Body} & \text{Weight} \end{array}$$ (5)

$$\text{DPSI}=\sqrt{\frac{\sum {\left(0-\text{GRFx}\right)}^{2}+\sum {\left(0-\text{GRFy}\right)}^{2}+\sum {\left(\text{BW}-\text{GRFz}\right)}^{2}}{\begin{array}{cc} \text{Number} & \begin{array}{ccc} \text{of} & \text{data} & \text{points} \end{array} \end{array}}}÷\begin{array}{cc} \text{Body} & \text{Weight} \end{array}$$ (6)

BW represents body weight, and GRF_x_, GRF_y_, and GRF_z_ represent the anteroposterior, mediolateral, and vertical ground reaction force components, respectively.

Whole-body COM velocity was calculated in Visual3D using a full-body segment model and extracted at IC, PKF, and TO. Braking-phase ΔCOM was calculated as the difference between velocity at IC and PKF (Equation 7), whereas recovery-phase ΔCOM velocity was calculated as the difference between velocity at PKF and TO (Equation 8). Joint angles were calculated using an XYZ Cardan sequence in the local joint coordinate system, representing sagittal-, frontal-, and transverse-plane rotations, respectively. Joint angles were expressed as signed rotations. For the racket-side limb, positive rotations represented hip flexion, adduction, and internal rotation; knee extension, varus, and internal rotation; and ankle dorsiflexion, inversion, and internal rotation. Negative values represented the corresponding opposite motions (hip extension, abduction, and external rotation; knee flexion, valgus, and external rotation; and ankle plantarflexion, eversion, and external rotation).

$$\Delta V_{\text{braking}}=V_{\text{PKF}}-V_{\text{IC}}$$ (7)

$$\Delta V_{\text{recovery}}=V_{\text{TO}}-V_{\text{PKF}}$$ (8)

Net internal joint moments were calculated using inverse dynamics and expressed in the local joint coordinate system. Positive moments represented hip flexion, adduction, and internal rotation; knee extension, varus, and internal rotation; and ankle dorsiflexion, inversion, and internal rotation, with negative values representing the corresponding opposite moments. Phase-specific sagittal-plane joint quasi-stiffness was estimated using a range-based approach as the ratio of the peak-to-peak change in normalized net joint moment to the peak-to-peak joint angular excursion within the same movement phase (Maloney and Fletcher, 2021; Xie et al., 2025a; Cai et al., 2026). This calculation was performed separately for the braking phase (initial contact to peak knee flexion) and recovery phase (peak knee flexion to toe-off). Vertical and anteroposterior GRF impulses were calculated using trapezoidal integration (Nielsen et al., 2020; Huang et al., 2025; Mulloy et al., 2021).

The MLSI and APSI represented the variability of the mediolateral and anteroposterior GRF components around their respective baseline values, whereas the VSI reflected deviations in vertical GRF from body weight. The DPSI integrated these three directional measures into a single index, with larger values denoting poorer dynamic postural stability (Wikstrom et al., 2007; Maeda et al., 2016). All stability indices were calculated during the braking phase, and GRF data within this phase were time-normalized to 101 points before analysis to account for potential differences in braking-phase duration between conditions.

Five characteristic events were identified from the vertical GRF profile: initial impact, secondary impact, amortization, weight acceptance, and drive-off (Kuntze et al., 2010; Nielsen et al., 2020) (see Figure 2). All kinetic variables were normalized to each participant’s body weight (BW). For each variable and condition, the mean of the three valid trials was used for analysis.

**FIGURE 2:**
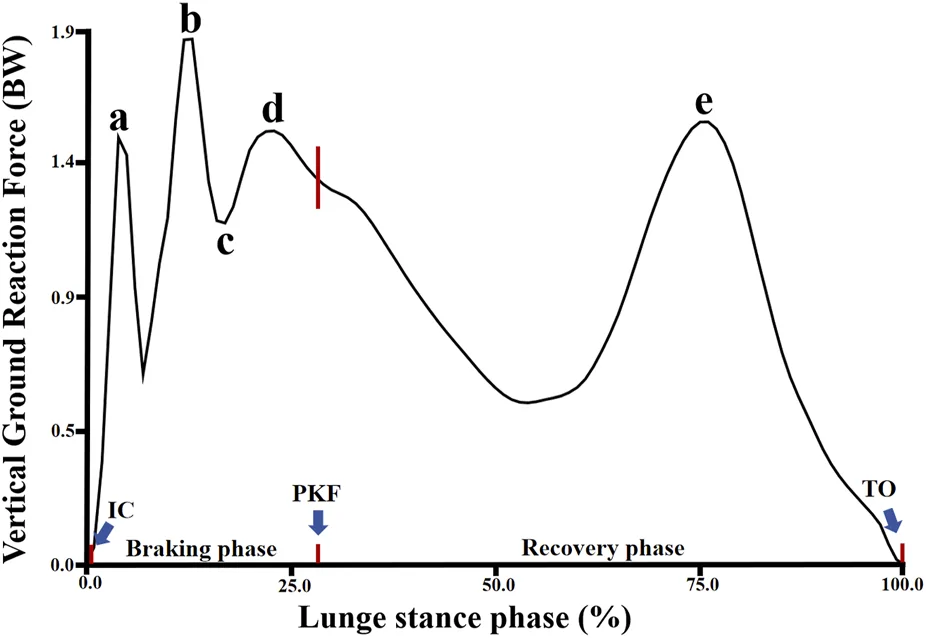
Example identification of events from the vertical ground reaction force curve during the badminton lunge stance phase. a: initial impact peak; b: secondary impact peak; c: amortization; d: weight acceptance; e: drive-off peak. IC: initial contact; PKF: peak knee flexion; TO: toe-off. The intervals IC–PKF, PKF–TO, and IC–TO represent the braking, recovery, and stance phases, respectively.

### 2.5 Statistical analysis

Data are presented as mean ± standard deviation. Residual normality was assessed using the Shapiro–Wilk test. A 2 × 2 repeated-measures ANOVA examined the main effects of fatigue condition (pre-fatigue vs. post-fatigue), lunge direction (forehand vs. backhand), and their interaction.

To control for multiple testing, the multiplicity structure was defined before inspection of the statistical results. Outcomes were grouped into five biologically related domains: (1) temporal and COM velocity outcomes; (2) joint kinematic outcomes; (3) external kinetic outcomes; (4) joint mechanical outcomes; and (5) dynamic postural stability outcomes. Within each biomechanical domain, the fatigue main effect, lunge-direction main effect, and fatigue × direction interaction were treated as separate inferential families because they addressed distinct inferential questions: overall fatigue-related changes, overall directional differences, and whether the effect of fatigue differed by lunge direction, respectively. This resulted in 15 prespecified inferential families (five biomechanical domains × three ANOVA effects). The five biomechanical domains were retained as separate families because they represented conceptually distinct biomechanical constructs. Accordingly, the Benjamini–Hochberg (BH) false discovery rate procedure (Benjamini and Hochberg, 1995) was applied separately to the p values for each ANOVA effect within each biomechanical domain. BH-adjusted p values below 0.05 were used for the primary statistical inference.

Because biomechanical outcomes within an inferential family may be correlated and the exact dependence structure could not be established, a sensitivity analysis was additionally performed using the more conservative Benjamini–Yekutieli (BY) procedure (Benjamini and Yekutieli, 2001) while retaining the same prespecified family structure. Unadjusted, BH-adjusted, and BY-adjusted p values for all outcomes and effects are reported in Supplementary Table S1.

To characterize the statistical sensitivity of the achieved sample, a design-based sensitivity analysis was performed for the fatigue main effect, lunge-direction main effect, and fatigue × direction interaction. Because each effect represents a one-degree-of-freedom within-subject contrast, sensitivity was expressed as the standardized within-subject effect size (dz). With n = 24, a two-sided α = 0.05, and 80% power, the minimum detectable effect was approximately dz = 0.60. These sensitivity calculations were based on the nominal α level and did not incorporate the subsequent FDR adjustment. Because this analysis was expressed in terms of one-degree-of-freedom within-subject contrasts, dz is not directly comparable with the Cohen’s f used in the original omnibus sample-size calculation.

## 3 Results

The fatigue protocol lasted 14.38 ± 2.82 min. At the end of the fatigue protocol, participants reached a heart rate of 183.56 ± 8.04 beats·min^-1^ and an RPE of 19.04 ± 0.98. The post-fatigue lunge-testing period lasted 1.28 ± 0.32 min, including repeated attempts required when trials did not meet the predefined validity criteria. The jump-height criterion corresponded to 47.00 ± 5.07 cm, representing 70% of individual maximal jump height. At the completion of post-fatigue lunge testing, heart rate and RPE were 169.08 ± 10.23 beats·min^-1^ and 17.68 ± 0.69, respectively. Although both measures decreased relative to protocol termination, they remained high at the end of the brief post-fatigue assessment period.

No statistically significant fatigue × direction interactions were detected after BH correction for any biomechanical outcome. The sensitivity analysis indicated that, with n = 24, 80% power corresponded to a standardized within-subject effect of approximately dz = 0.60; a conventional medium standardized within-subject effect (dz = 0.50) corresponded to approximately 65% power. Fatigue shortened the braking phase duration (0.21 ± 0.04 vs. 0.19 ± 0.03 s; *p*_BH_ < 0.01; ηp^2^ = 0.35), reduced the magnitude of anteroposterior velocity at TO (−1.02 ± 0.23 vs. −0.84 ± 0.21 m/s; *p*_BH_ < 0.01; ηp^2^ = 0.62), and reduced braking change (1.21 ± 0.20 vs. 1.09 ± 0.19 m/s; *p*_BH_ < 0.03; ηp^2^ = 0.23) and recovery-phase (1.40 ± 0.30 vs. 1.25 ± 0.30 m/s; *p*_BH_ < 0.01; ηp^2^ = 0.47) change in anteroposterior COM velocity. Backhand lunges showed lower COM velocity at initial contact (1.47 ± 0.21 vs. 1.61 ± 0.18 m/s; *p*_BH_ <0.01; ηp^2^ = 0.56) and a smaller recovery-phase velocity change than forehand lunges (1.26 ± 0.28 vs. 1.39 ± 0.31 m/s; *p*_BH_ < 0.01; ηp^2^ = 0.43) (Table 1).

**TABLE 1:** Phase durations and anteroposterior COM velocity during badminton lunges (mean ± SD).

Variables	Pre		Post		Fatigue	Direction	Interaction
	Backhand	Forehand	Backhand	Forehand	*p*_BH_ (ηp^2^)	*p*_BH_ (ηp^2^)	*p*_BH_ (ηp^2^)
Phase durations (s)							
Braking phase	0.20 ± 0.03	0.22 ± 0.04	0.18 ± 0.03	0.19 ± 0.03	**< 0.01 (0.35)**	0.05 (0.21)	0.69 (0.07)
Recovery phase	0.42 ± 0.16	0.40 ± 0.15	0.38 ± 0.18	0.39 ± 0.18	0.23 (0.06)	0.70 (0.01)	0.69 (0.06)
Stance phase	0.62 ± 0.16	0.62 ± 0.15	0.57 ± 0.18	0.58 ± 0.18	**0.05(0.18)**	0.70 (0.01)	0.69 (0.03)
Anteroposterior COM velocity (m/s)							
Velocity at IC	1.52 ± 0.25	1.64 ± 0.21	1.42 ± 0.17	1.58 ± 0.16	0.07 (0.14)	**< 0.01 (0.56)**	0.70 (0.02)
Velocity at TO	−1.00 ± 0.21	−1.04 ± 0.24	−0.83 ± 0.17	−0.86 ± 0.24	**< 0.01(0.62)**	0.46 (0.04)	0.80 (<0.01)
Braking-phase ΔCOM velocity	1.18 ± 0.22	1.23 ± 0.19	1.08 ± 0.17	1.11 ± 0.21	**0.03 (0.23)**	0.17 (0.11)	0.75 (0.01)
Recovery-phase ΔCOM velocity	1.34 ± 0.29	1.45 ± 0.3	1.17 ± 0.27	1.32 ± 0.33	**< 0.01 (0.47)**	**< 0.01 (0.43)**	0.69 (0.03)

Values are mean ± SD., the braking, recovery, and stance phases were defined as IC–PKF, PKF–TO, and IC–TO, respectively. IC, initial contact; PKF, peak knee flexion; TO, toe-off; COM, center of mass. Positive COM, velocity indicates movement toward the net. pBH, Benjamini–Hochberg-adjusted p value; ηp^2^, partial eta squared. Statistical significance was determined using unrounded pBH, values; displayed p values are rounded to two decimal places. Significant pBH, values are bolded.

Fatigue-related kinematic changes were characterized by reduced lower-limb joint excursions across multiple planes. Fatigue reduced peak hip flexion (83.22 ± 14.46 vs. 71.16° ± 17.36**°**; *p*_BH_ < 0.01; ηp^2^ = 0.65) and abduction (−47.46 ± 7.70 vs. −42.24° ± 8.50**°**; *p*_BH_ < 0.01; ηp^2^ = 0.55), knee flexion (−71.25 ± 10.05 vs. −66.33° ± 9.38**°**; *p*_BH_ = 0.03; ηp^2^ = 0.23) and varus (30.01 ± 8.45 vs. 24.75° ± 9.53**°**; *p*_BH_ < 0.01; ηp^2^ = 0.40), ankle dorsiflexion (16.78 ± 5.60 vs. 14.50° ± 5.15**°**; *p*_BH_ = 0.02; ηp^2^ = 0.25), hip sagittal-plane (46.72 ± 12.48 vs. 34.36° ± 9.57**°**; *p*_BH_ < 0.01; ηp^2^ = 0.66) and transverse-plane (31.24 ± 9.61 vs. 23.70° ± 7.78**°**; *p*_BH_ < 0.01; ηp^2^ = 0.52) ROM, and knee sagittal-plane (63.43 ± 10.86 vs. 57.25° ± 8.17**°**; *p*_BH_ < 0.01; ηp^2^ = 0.34) and frontal-plane (26.43 ± 7.90 vs. 21.61° ± 8.21**°**; *p*_BH_ < 0.01; ηp^2^ = 0.42) ROM, but increased peak hip external rotation (−16.09 ± 10.83 vs. −22.62° ± 13.85**°**; *p*_BH_ < 0.01; ηp^2^ = 0.35).

Compared with forehand lunges, backhand lunges showed smaller hip abduction (−42.26 ± 7.52 vs. −47.14° ± 8.69**°**; *p*_BH_ < 0.01; ηp^2^ = 0.46) and external rotation (−16.06 ± 12.33 vs. −22.65° ± 12.35**°**; *p*_BH_ < 0.01; ηp^2^ = 0.74), greater knee flexion (−70.21 ± 9.40 vs. −67.37° ± 10.03**°**; *p*_BH_ < 0.01; ηp^2^ = 0.42), less knee varus (25.35 ± 8.38 vs. 29.41° ± 9.59**°**; *p*_BH_ < 0.01; ηp^2^ = 0.34), greater ankle dorsiflexion (16.76 ± 5.51 vs. 14.53° ± 5.24**°**; *p*_BH_ < 0.01; ηp^2^ = 0.41) and inversion (14.18 ± 5.82 vs. 10.23° ± 4.82**°**; *p*_BH_ < 0.01; ηp^2^ = 0.49), smaller knee transverse-plane ROM (26.54 ± 7.08 vs. 29.33° ± 9.19**°**; *p*_BH_ = 0.03; ηp^2^ = 0.25), and greater ankle sagittal-plane ROM (34.19 ± 6.87 vs. 31.80° ± 6.80**°**; *p*_BH_ = 0.01; ηp^2^ = 0.34). The pre- and post-fatigue profiles of the outcomes with the largest significant fatigue and lunge-direction main effects are illustrated in Figure 3. No fatigue × direction interaction reached statistical significance after BH correction (Table 2).

**FIGURE 3:**
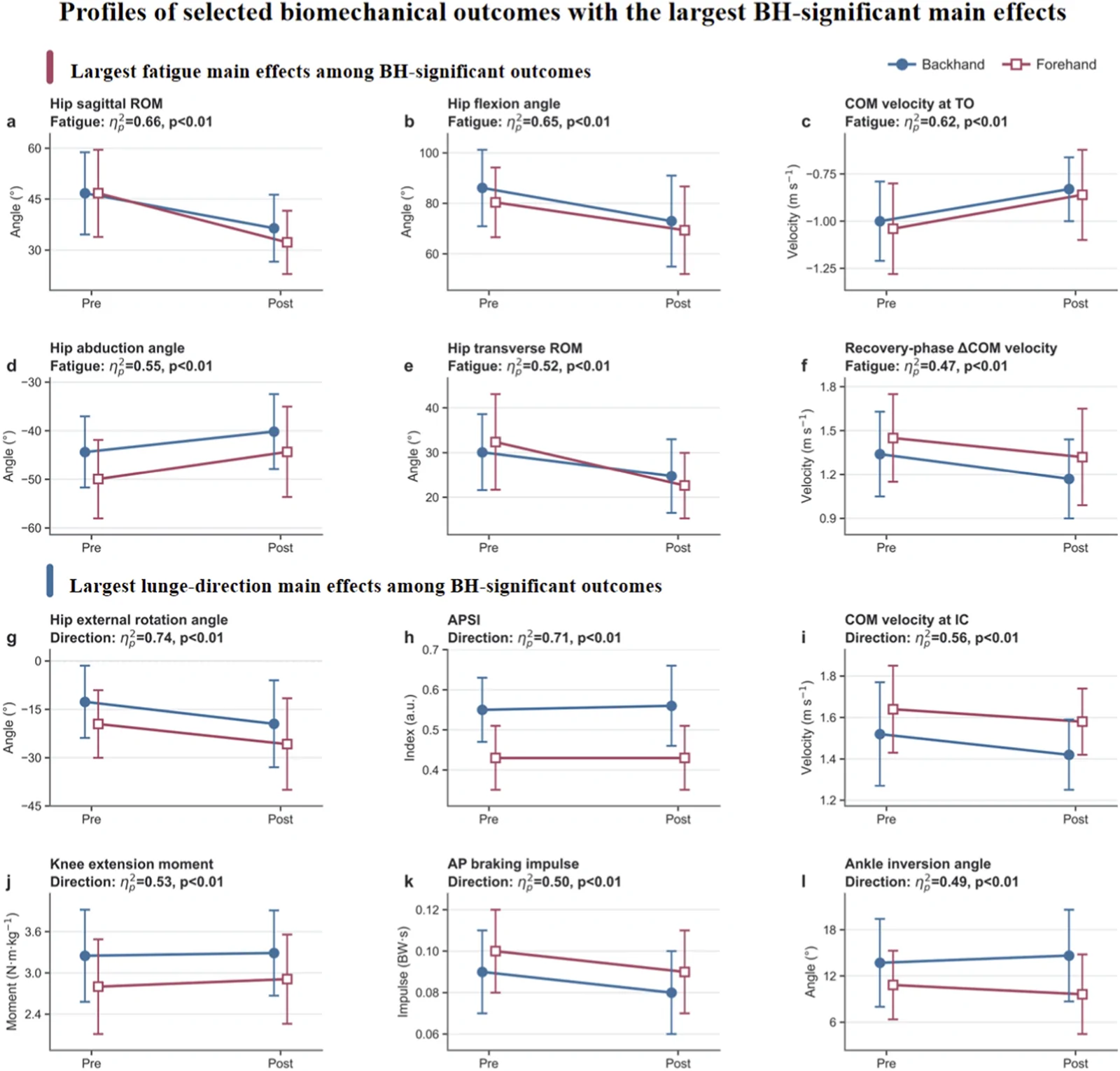
Pre- and post-fatigue profiles of selected biomechanical outcomes with the largest significant fatigue and lunge-direction main effects after BH correction. Panels **(a–f)** show fatigue effects, and panels **(g–l)** show direction effects. Values are mean ± SD; ***p***_BH_ denotes the Benjamini–Hochberg-adjusted p value. AP, anterior–posterior; APSI, anterior–posterior stability index; COM, center of mass; IC, initial contact; ROM, range of motion; TO, toe-off.

**TABLE 2:** Peak lower-limb joint angles and ranges of motion during the stance phase of the badminton lunge (mean ± SD).

Variables		Pre		Post		Fatigue	Direction	Interaction
		Backhand	Forehand	Backhand	Forehand	*p*_BH_ (ηp^2^)	*p*_BH_ (ηp^2^)	*p*_BH_ (ηp^2^)
Joint peak angle during stance phase (°)								
Hip	Flexion	86.09 ± 15.15	80.36 ± 13.78	72.96 ± 17.96	69.36 ± 17.30	**<0.01 (0.65)**	0.14 (0.12)	1.00 (0.01)
	Abduction	−44.37 ± 7.33	−49.95 ± 8.08	−40.15 ± 7.70	−44.33 ± 9.30	**<0.01 (0.55)**	**<0.01 (0.46)**	0.93 (0.02)
	External rotation	−12.65 ± 11.19	−19.53 ± 10.46	−19.47 ± 13.48	−25.78 ± 14.23	**<0.01 (0.35)**	**<0.01 (0.74)**	1.00 (0.01)
Knee	Flexion	−72.48 ± 9.67	−70.02 ± 10.43	−67.94 ± 9.13	−64.72 ± 9.62	**0.03 (0.23)**	**<0.01 (0.42)**	0.93 (0.02)
	Varus	28.01 ± 8.37	32.02 ± 8.53	22.70 ± 8.40	26.81 ± 10.66	**<0.01 (0.40)**	**0.01 (0.34)**	1.00 (<0.01)
	External rotation	−33.57 ± 14.82	−35.86 ± 14.45	−30.51 ± 12.08	−35.21 ± 14.44	0.31 (0.06)	0.06 (0.19)	0.78 (0.05)
Ankle	Dorsiflexion	17.90 ± 5.34	15.67 ± 5.87	15.61 ± 5.69	13.39 ± 4.60	**0.02 (0.25)**	**<0.01 (0.41)**	1.00 (<0.01)
	Inversion	13.71 ± 5.70	10.83 ± 4.46	14.65 ± 5.95	9.63 ± 5.17	0.88 (<0.01)	**<0.01 (0.49)**	0.52 (0.09)
	External rotation	−12.44 ± 12.86	−10.75 ± 12.27	−10.55 ± 9.24	−11.33 ± 9.64	0.86 (<0.01)	0.85 (<0.01)	0.93 (0.02)
Joint range of motion during stance phase (°)								
Hip	Sagittal	46.72 ± 12.11	46.72 ± 12.84	36.45 ± 9.84	32.28 ± 9.29	**< 0.01 (0.66)**	0.18 (0.10)	0.35 (0.15)
	Coronal	23.00 ± 7.22	25.81 ± 7.13	20.54 ± 6.95	23.35 ± 7.68	0.07 (0.17)	0.07 (0.17)	1.00 (<0.01)
	Transverse	30.10 ± 8.51	32.38 ± 10.70	24.76 ± 8.23	22.63 ± 7.33	**< 0.01 (0.52)**	0.94 (<0.01)	0.28 (0.21)
Knee	Sagittal	63.78 ± 10.77	63.07 ± 10.96	57.71 ± 6.90	56.79 ± 9.44	**< 0.01 (0.34)**	0.44 (0.04)	1.00 (<0.01)
	Coronal	25.1 ± 7.95	27.76 ± 7.86	20.16 ± 7.55	23.06 ± 8.88	**< 0.01 (0.42)**	0.07 (0.17)	1.00 (<0.01)
	Transverse	26.04 ± 6.16	29.84 ± 9.99	27.03 ± 8.00	28.82 ± 8.39	0.99 (<0.01)	**0.03 (0.25)**	0.78 (0.05)
Ankle	Sagittal	35.46 ± 7.38	31.93 ± 6.31	32.92 ± 6.37	31.68 ± 7.28	0.20 (0.09)	**0.01 (0.34)**	0.52 (0.10)
	Coronal	17.40 ± 6.40	19.58 ± 7.00	19.88 ± 7.95	19.37 ± 5.87	0.20 (0.10)	0.58 (0.02)	0.28 (0.19)
	Transverse	14.87 ± 4.74	13.88 ± 7.33	16.13 ± 4.92	14.64 ± 5.25	0.52 (0.03)	0.18 (0.09)	1.00 (<0.01)

Values are mean ± SD., the braking, recovery, and stance phases were defined as IC–PKF, PKF–TO, and IC–TO, respectively. IC, initial contact; PKF, peak knee flexion; TO, toe-off; ROM, range of motion. pBH, Benjamini–Hochberg-adjusted p value; ηp^2^, partial eta squared. Statistical significance was determined using unrounded pBH, values; displayed p values are rounded to two decimal places. Significant pBH, values are bolded.

Fatigue altered impact attenuation characteristics by reducing impact-related force impulses while increasing weight-acceptance vertical GRF. Fatigue reduced the initial vertical impact peak (1.75 ± 0.45 vs. 1.53 ± 0.33 BW; *p*_BH_ < 0.01; ηp^2^ = 0.37) and anteroposterior (braking phase: 0.10 ± 0.02 vs. 0.08 ± 0.02 BW·s; *p*_BH_ = 0.01; ηp^2^ = 0.33; recovery phase: 0.15 ± 0.03 vs. 0.13 ± 0.03 BW·s; *p*_BH_ < 0.01; ηp^2^ = 0.43) and vertical (braking phase: 0.28 ± 0.04 vs. 0.25 ± 0.04 BW·s; *p*_BH_ < 0.01; ηp^2^ = 0.42; recovery phase: 0.42 ± 0.11 vs. 0.38 ± 0.12 BW·s; *p*_BH_ = 0.04; ηp^2^ = 0.20) impulses during both phases, but increased vertical GRF at weight acceptance (1.63 ± 0.19 vs. 1.69 ± 0.18 BW; *p*_BH_ = 0.04; ηp^2^ = 0.21). Backhand lunges showed greater secondary-impact (1.82 ± 0.24 vs. 1.65 ± 0.24 BW; *p*_BH_ < 0.01; ηp^2^ = 0.47) and amortization vertical GRFs (1.50 ± 0.26 vs. 1.40 ± 0.25 BW; *p*_BH_ < 0.01; ηp^2^ = 0.41), lower anteroposterior braking impulse (0.08 ± 0.02 vs. 0.10 ± 0.02 BW·s; *p*_BH_ < 0.01; ηp^2^ = 0.50), higher DPSI (0.99 ± 0.15 vs. 0.91 ± 0.14; *p*_BH_ < 0.01; ηp^2^ = 0.41), MLSI (0.56 ± 0.09 vs. 0.43 ± 0.08; *p*_BH_ < 0.01; ηp^2^ = 0.71), and VSI (0.64 ± 0.14 vs. 0.58 ± 0.13; *p*_BH_ < 0.01; ηp^2^ = 0.32), and lower APSI (0.50 ± 0.07 vs. 0.54 ± 0.07; *p*_BH_ < 0.01; ηp^2^ = 0.42) than forehand lunges. No fatigue × direction interaction reached statistical significance after BH correction (Table 3).

**TABLE 3:** Ground reaction force, impulse, and dynamic postural stability outcomes during badminton lunges (mean ± SD).

Variables		Pre		Post		Fatigue	Direction	Interaction
		Backhand	Forehand	Backhand	Forehand	*p*_BH_ (ηp^2^)	*p*_BH_ (ηp^2^)	*p*_BH_ (ηp^2^)
Vertical ground reaction force (BW)								
Initial impact peak		1.77 ± 0.42	1.74 ± 0.48	1.58 ± 0.29	1.47 ± 0.36	**<0.01** (0.37)	0.26 (0.10)	0.48 (0.05)
Secondary impact peak		1.87 ± 0.24	1.68 ± 0.23	1.76 ± 0.24	1.62 ± 0.25	0.05 (0.17)	**<0.01** (0.47)	0.58 (0.02)
Amortization		1.52 ± 0.25	1.42 ± 0.24	1.47 ± 0.27	1.38 ± 0.26	0.40 (0.04)	**<0.01** (0.41)	0.93 (<0.01)
Weight acceptance		1.63 ± 0.20	1.63 ± 0.18	1.72 ± 0.19	1.68 ± 0.17	**0.04** (0.21)	0.42 (0.05)	0.48 (0.04)
Drive-off		1.56 ± 0.26	1.53 ± 0.22	1.52 ± 0.35	1.53 ± 0.32	0.70 (0.01)	0.90 (<0.01)	0.58 (0.02)
Impulse (BW·s)								
Anteroposterior impulse	Braking	0.09 ± 0.02	0.1 ± 0.02	0.08 ± 0.02	0.09 ± 0.02	**0.01** (0.33)	**< 0.01** (0.50)	0.48 (0.05)
	Recovery	0.15 ± 0.03	0.15 ± 0.03	0.13 ± 0.03	0.14 ± 0.03	**< 0.01** (0.39)	0.42 (0.06)	0.48 (0.06)
Vertical impulse	Braking	0.28 ± 0.03	0.29 ± 0.04	0.25 ± 0.03	0.25 ± 0.04	**< 0.01** (0.43)	0.42 (0.03)	0.48 (0.09)
	Recovery	0.43 ± 0.12	0.41 ± 0.10	0.38 ± 0.12	0.38 ± 0.12	**0.04** (0.20)	0.42 (0.04)	0.48 (0.11)
Dynamic postural stability index at braking phase								
DPSI		0.99 ± 0.15	0.90 ± 0.15	0.99 ± 0.15	0.91 ± 0.13	0.77 (<0.01)	**<0.01** (0.41)	0.75 (0.02)
MLSI		0.55 ± 0.08	0.43 ± 0.08	0.56 ± 0.10	0.43 ± 0.08	0.77 (<0.01)	**<0.01** (0.71)	0.93 (<0.01)
APSI		0.50 ± 0.08	0.54 ± 0.07	0.49 ± 0.06	0.54 ± 0.06	0.77 (<0.01)	**<0.01** (0.42)	0.75 (0.02)
VSI		0.63 ± 0.15	0.57 ± 0.14	0.64 ± 0.13	0.59 ± 0.12	0.77 (<0.01)	**<0.01** (0.32)	0.75 (0.02)

Values are mean ± SD., the braking, recovery, and stance phases were defined as IC–PKF, PKF–TO, and IC–TO, respectively. IC, initial contact; PKF, peak knee flexion; TO, toe-off; BW, body weight; DPSI, dynamic postural stability index; APSI, anteroposterior stability index; MLSI, mediolateral stability index; VSI, vertical stability index. pBH, Benjamini–Hochberg-adjusted p value; ηp^2^, partial eta squared. Statistical significance was determined using unrounded pBH, values; displayed p values are rounded to two decimal places. Significant pBH, values are bolded.

Fatigue reduced peak hip extension moment (−4.17 ± 1.57 vs. −3.62 ± 1.18 N·m·kg^-1^; *p*_BH_ = 0.04; ηp^2^ = 0.27) and increased recovery-phase hip (0.08 ± 0.05 vs. 0.10 ± 0.05 N·m·kg^−1^·deg^−1^; *p*_BH_ < 0.01; ηp^2^ = 0.41) and knee quasi-stiffness (0.05 ± 0.01 vs. 0.06 ± 0.02 N·m·kg^−1^·deg^−1^; *p*_BH_ < 0.01; ηp^2^ = 0.44). Backhand lunges showed greater peak knee extension (3.27 ± 0.64 vs. 2.86 ± 0.67 N·m·kg^-1^; *p*_BH_ < 0.01; ηp^2^ = 0.53) and ankle dorsiflexion moments (0.99 ± 0.26 vs. 0.89 ± 0.22 N·m·kg^-1^; *p*_BH_ = 0.04; ηp^2^ = 0.29) than forehand lunges. No other main effects or fatigue × direction interactions reached statistical significance after BH correction (Table 4). The distribution and magnitude of fatigue, lunge-direction, and interaction effects across all outcomes are summarized in Figure 4.

**TABLE 4:** Peak lower-limb joint moments and joint quasi-stiffness during badminton lunges (mean ± SD).

Variables		Pre		Post		Fatigue	Direction	Interaction
		Backhand	Forehand	Backhand	Forehand	*p*_BH_ (ηp^2^)	*p*_BH_ (ηp^2^)	*p*_BH_ (ηp^2^)
Peak joint moments during stance phase (N·m·kg^-1^)								
Hip	Extension	−4.18 ± 1.54	−4.17 ± 1.61	−3.70 ± 1.15	−3.54 ± 1.21	**0.04 (0.27)**	0.62 (0.01)	0.89 (0.01)
	Adduction	4.61 ± 1.75	5.02 ± 2.02	3.88 ± 1.22	4.21 ± 1.77	0.07 (0.21)	0.28 (0.10)	0.92 (<0.01)
	Internal rotation	1.80 ± 1.23	1.33 ± 0.69	1.43 ± 0.59	1.33 ± 0.72	0.26 (0.09)	0.13 (0.20)	0.89 (0.09)
Knee	Extension	3.25 ± 0.67	2.80 ± 0.69	3.29 ± 0.62	2.91 ± 0.65	0.58 (0.02)	**<0.01 (0.53)**	0.89 (0.02)
	Varus	0.71 ± 0.35	0.69 ± 0.34	0.60 ± 0.29	0.55 ± 0.28	0.06 (0.23)	0.43 (0.04)	0.89 (0.03)
	Internal rotation	0.77 ± 0.34	0.82 ± 0.26	0.72 ± 0.25	0.81 ± 0.31	0.58 (0.02)	0.35 (0.07)	0.89 (0.01)
Ankle	Dorsiflexion	1.03 ± 0.31	0.93 ± 0.26	0.95 ± 0.22	0.85 ± 0.19	0.08 (0.18)	**0.04 (0.29)**	0.89 (<0.01)
	Inversion	0.34 ± 0.17	0.28 ± 0.16	0.36 ± 0.21	0.31 ± 0.14	0.48 (0.05)	0.20 (0.13)	0.89 (0.01)
	Internal rotation	0.13 ± 0.07	0.12 ± 0.06	0.14 ± 0.06	0.12 ± 0.05	0.58 (0.01)	0.20 (0.13)	0.89 (0.01)
Joint quasi-stiffness (N·m·kg^−1^·deg^−1^)								
Hip	Braking	0.272 ± 0.12	0.234 ± 0.12	0.290 ± 0.09	0.268 ± 0.11	0.14 (0.14)	0.19 (0.16)	0.89 (0.02)
	Recovery	0.077 ± 0.05	0.073 ± 0.04	0.098 ± 0.05	0.098 ± 0.05	**<0.01 (0.41)**	0.66 (0.01)	0.92 (<0.01)
Knee	Braking	0.088 ± 0.02	0.087 ± 0.02	0.088 ± 0.02	0.094 ± 0.02	0.49 (0.04)	0.35 (0.07)	0.89 (0.13)
	Recovery	0.049 ± 0.01	0.047 ± 0.01	0.059 ± 0.01	0.056 ± 0.02	**<0.01 (0.44**)	0.35 (0.06)	0.92 (<0.01)
Ankle	Braking	0.070 ± 0.02	0.068 ± 0.02	0.069 ± 0.02	0.065 ± 0.02	0.58 (0.02)	0.35 (0.06)	0.89 (0.02)
	Recovery	0.056 ± 0.01	0.057 ± 0.01	0.057 ± 0.02	0.053 ± 0.02	0.58 (0.02)	0.57 (0.02)	0.89 (0.04)

Values are mean ± SD., the braking, recovery, and stance phases were defined as IC–PKF, PKF–TO, and IC–TO, respectively. IC, initial contact; PKF, peak knee flexion; TO, toe-off. Joint quasi-stiffness was calculated separately for the braking and recovery phases. pBH, Benjamini–Hochberg-adjusted p value; ηp^2^, partial eta squared. Statistical significance was determined using unrounded pBH, values; displayed p values are rounded to two decimal places. Significant pBH, values are bolded. Joint moments are net internal moments. Positive values represent hip flexion, adduction, and internal rotation; knee extension, varus, and internal rotation; and ankle dorsiflexion, inversion, and internal rotation; negative values represent the opposite directions.

**FIGURE 4:**
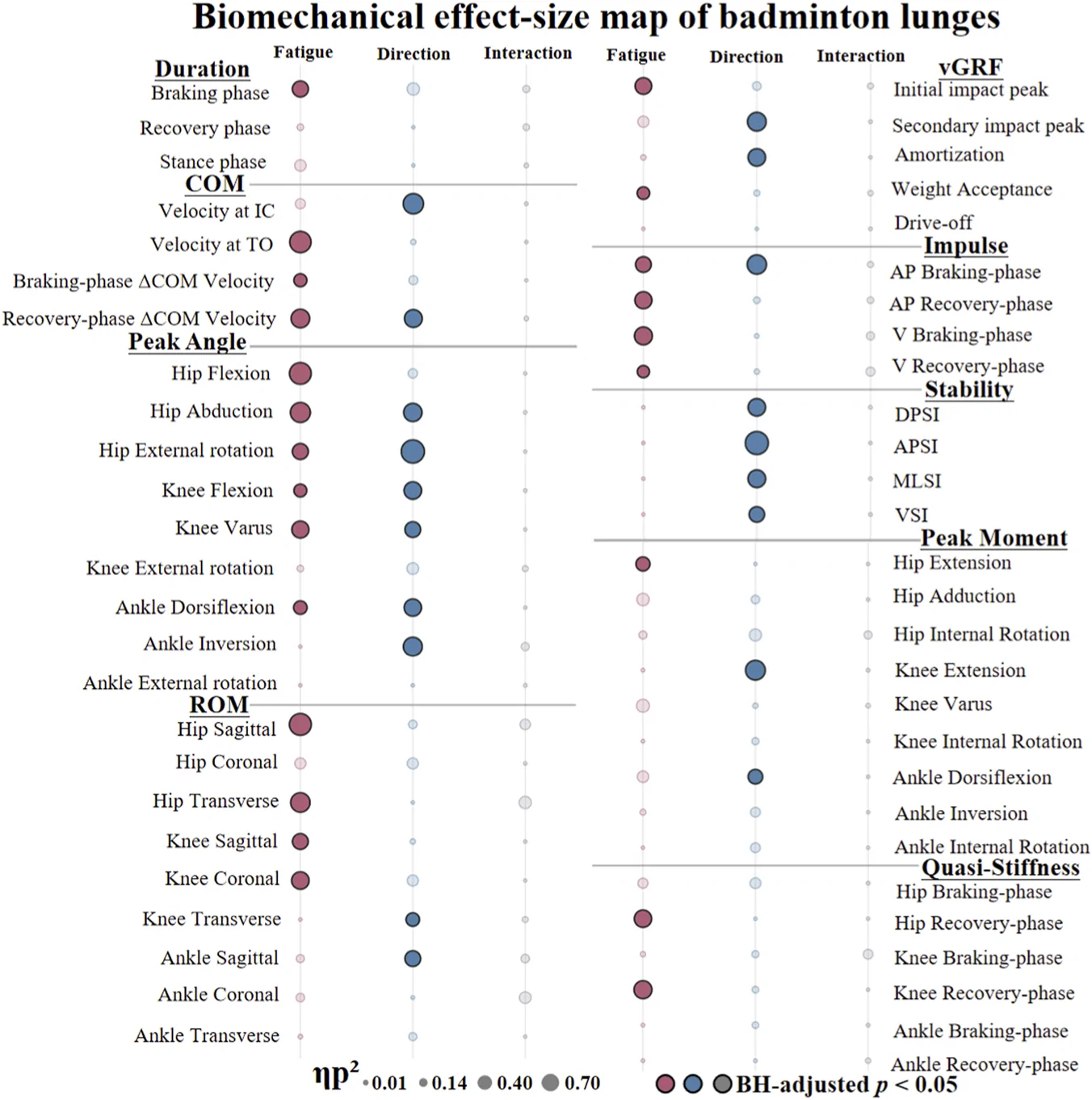
Biomechanical effect-size map of badminton lunges. Red, blue, and gray circles represent the fatigue main effect, lunge-direction main effect, and fatigue × direction interaction, respectively. Circle size is proportional to partial eta squared (ηp^2^), with larger circles indicating larger effect sizes; reference sizes of ηp^2^ = 0.01, 0.14, 0.40, and 0.70 are shown. Dark filled circles with black outlines indicate effects that remained statistically significant after Benjamini–Hochberg correction (pBH <0.05), whereas lighter circles indicate non-significant effects.

The BY sensitivity analysis showed that nine effects that were significant under the primary BH procedure did not remain significant under the more conservative BY procedure: the fatigue effects on stance duration, braking-phase anteroposterior COM velocity change, knee flexion angle, ankle dorsiflexion angle, weight-acceptance GRF, recovery-phase vertical impulse, and hip extension moment, as well as the lunge-direction effects on knee transverse-plane ROM and ankle dorsiflexion moment. All fatigue × direction interactions remained non-significant under both procedures (Supplementary Table S1).

## 4 Discussion

This study examined the independent and interactive effects of fatigue and lunge direction on racket-side lower-limb biomechanics during badminton lunges. Fatigue and lunge direction showed significant main effects on selected racket-side lower-limb biomechanical outcomes. However, given the limited statistical sensitivity for interaction effects, these findings should not be interpreted as evidence that the effects of fatigue were independent of lunge direction. Specifically, fatigue shortened the braking phase, reduced multiplanar joint motion, COM velocity changes, braking impulses, and recovery-phase anteroposterior impulse, while increasing recovery-phase hip and knee quasi-stiffness. These findings indicate a more constrained braking strategy and reduced capacity to restore movement after lunging. Lunge direction primarily affected impact loading and dynamic postural stability, with backhand lunges showing greater secondary-impact loading, greater overall, mediolateral, and vertical stability index values, a greater knee extension moment, and greater ankle motion and stability-control demands.

Lunge-phase duration has been considered an important temporal indicator of badminton lunge performance, reflecting players’ ability to reach the shuttlecock and return to the base position in time (Lee and Loh, 2019; Lam et al., 2020). Previous studies have suggested that reduced sagittal-plane joint motion, particularly smaller hip flexion and limited forward displacement of the knee beyond the toes, may facilitate a quicker return after lunging (Huang et al., 2014; Mei et al., 2017). In the present study, fatigue shortened braking-phase duration and reduced sagittal-plane motion, particularly hip and knee ROM, indicating a more constrained lower-limb movement strategy. However, because recovery-phase duration was not significantly altered, the robust reduction in braking-phase duration is more appropriately interpreted as an alteration in braking strategy rather than evidence of improved repositioning performance.

Fatigue also reduced peak hip abduction, peak knee varus angle, and coronal-plane ROM of the knee, suggesting a more constrained movement strategy in both sagittal and frontal planes. These findings are also partly consistent with those reported in amateur badminton players, with Xie et al. (2025a) reporting a reduction in coronal-plane knee ROM under fatigue. Previous studies have indicated that constrained coronal- and transverse-plane knee motion may adversely affect lunge performance in players with knee pain and players of different competitive levels (Huang et al., 2014; Lin et al., 2015). Thus, athletes may have shortened the braking phase under fatigue by restricting hip, knee, and ankle motion, but this strategy may reduce movement efficiency during subsequent repositioning and limit the capacity for rapid movement recovery. During badminton rallies, this reduced recovery capacity may hinder rapid return toward the base position after a lunge, potentially reducing readiness for subsequent strokes. Similar fatigue-related alterations in movement capacity have also been reported in other sporting contexts, with soccer-specific fatigue affecting flexibility, dynamic balance, and performance-related outcomes (Iranmanesh et al., 2025).

This interpretation is conceptually consistent with sprint and sprint-hurdle biomechanics research showing that impulse generation is closely related to locomotor velocity regulation. Previous sprint research has shown that greater propulsive impulse is associated with faster sprint velocities (Hunter et al., 2005), while sprint-hurdle evidence suggests that larger propulsive impulse during the recovery step contributes to the restoration of running speed (Nagahara et al., 2021). In the present study, fatigue reduced recovery-phase anteroposterior impulse, horizontal COM velocity at toe-off, and horizontal COM velocity gain during the recovery phase. Given that badminton lunges require players to decelerate after landing and subsequently reverse body motion toward the court center (Kuntze et al., 2010; Lee and Loh, 2019), these findings suggest that fatigue may impair the impulse-generating and velocity-increase capacity required for rapid repositioning after a lunge.

The badminton lunge is a closed-chain movement involving triple flexion and subsequent triple extension of the hip, knee, and ankle on the racket-side limb (Maloney, 2018). Consistent with previous studies, the present study showed greater sagittal-plane angular displacement at the knee than at the hip and ankle, highlighting the important role of the knee in decelerating the body during the lunge (Kuntze et al., 2010; Maloney, 2018; Xie et al., 2025a). Under fatigue, the initial impact peak GRF and knee sagittal-plane ROM were reduced, suggesting an altered impact-attenuation and knee movement strategy. Because ligament loading and joint contact forces were not directly assessed, these findings should not be interpreted as evidence of altered tissue-level loading. Future research integrating musculoskeletal modeling may provide further insight into knee and ligament loading patterns.

Fatigue also increased recovery-phase knee quasi-stiffness, indicating an altered phase-specific relationship between net joint moment and angular excursion. The badminton lunge forms part of a start–stop–recover cycle and can be interpreted within the framework of stretch-shortening cycle tasks (Komi, 2000; Kuntze et al., 2010; Maloney, 2018). Previous studies have suggested that sufficient lower-limb stiffness may contribute to elastic energy storage and force generation during stretch-shortening cycle activities, whereas excessive stiffness may increase mechanical demands around the knee joint (Tracy A, 2010; Valldecabres et al., 2018; Brazier et al., 2019). However, the present quasi-stiffness estimate should not be interpreted as a direct measure of joint impedance or tissue-level stiffness. Therefore, the reduction in knee sagittal-plane motion together with increased recovery-phase knee quasi-stiffness may suggest that fatigue alters knee mechanical-control strategies during badminton lunges. These changes indicate an altered phase-specific knee mechanical-control strategy; however, their relationship with injury-related mechanisms cannot be established from the present data. From a practical perspective, training programs emphasizing eccentric strength, lower-limb force production, and movement control under fatigue may help athletes maintain braking capacity and post-lunge recovery ability. Previous intervention research has shown that resistance and complex training can modify lower-limb biomechanical characteristics during badminton lunges, suggesting that targeted strength-based training may be a potential strategy to maintain more effective lunge mechanics and movement control (Xie et al., 2025c). Whether such training approaches can attenuate fatigue-related biomechanical alterations requires further investigation.

Forehand and backhand forward lunges represent two important directional lunge tasks in badminton biomechanics research (Hong et al., 2014; Xie et al., 2025b). In the present study, backhand lunges showed greater loading and stability-control demands than forehand lunges. Specifically, GRF at the secondary impact peak and during the amortization phase was greater in backhand lunges, consistent with Hong et al. (2014), who also reported higher GRFs during backhand forward lunges. Moreover, the Dynamic Postural Stability Index (DPSI) quantifies dynamic postural control during the transition from movement to a more stable state (Wikstrom et al., 2005; Maeda et al., 2016). Because badminton lunges also require rapid deceleration and stabilization during the braking phase, the present study used a braking-phase DPSI to assess dynamic postural stability in this task (Kuntze et al., 2010; Maloney, 2018). Backhand lunges showed greater braking-phase DPSI, MLSI, and VSI, whereas forehand lunges showed greater APSI. Because higher stability indices indicate poorer dynamic postural stability (Sell et al., 2013; Maeda et al., 2016), these findings suggest that backhand lunges may challenge overall, medial–lateral, and vertical force control to a greater extent, whereas forehand lunges may impose greater anterior–posterior stability demands.

The greater ankle dorsiflexion angle and sagittal-plane ROM observed in backhand lunges may reflect an ankle-based shock-absorption strategy to cope with greater GRF. Greater ankle sagittal-plane ROM may facilitate energy dissipation during impact (Xu et al., 2024), while larger dorsi-plantar flexion excursions may also indicate greater mechanical demands on the calf–ankle complex (Hensley and Paup, 1979; Fahlström et al., 1998; Fahlström et al., 2002; Mei et al., 2017). Backhand lunges also showed a greater peak ankle inversion angle, consistent with Xie et al. (2025a). Increased ankle inversion has been implicated in the biomechanical mechanisms of lateral ankle sprain (Fong et al., 2007; 2009; Fu et al., 2017). Thus, the ankle kinematic characteristics observed during backhand lunges are consistent with biomechanical features previously associated with ankle injury mechanisms; however, whether these differences are related to actual injury risk requires further investigation.

Regarding joint moments, the present study showed a greater knee extension moment in backhand lunges, partly consistent with Xie et al. (2025a), who reported greater peak knee extension moment during backhand lunges in amateur badminton players. Other direction-related joint moment differences reported by Xie et al. were not observed, possibly due to differences in playing level or motion-control strategies. The present study also found greater hip external rotation during forehand lunges, consistent with previous studies reporting direction-specific hip rotation strategies during badminton lunges (Valldecabres et al., 2018; 2022; Xie et al., 2025b). Together, these findings suggest that forehand and backhand lunges involve distinct lower-limb control strategies, with backhand lunges showing greater overall loading and ankle stability-control demands.

Several limitations should be acknowledged. First, participants were highly trained male badminton athletes, which may limit the generalizability of the findings to female players, recreational athletes, and athletes with different training backgrounds. Second, the laboratory fatigue protocol does not fully reproduce the complexity of match fatigue. Some recovery may also have occurred during post-fatigue testing, and the absence of baseline HR and RPE prevented quantification of changes relative to individual resting levels. Third, only the racket-side limb was analyzed; therefore, the findings may not fully represent bilateral lunge biomechanics. Fourth, statistical sensitivity was limited for effects below approximately dz = 0.60; therefore, the non-significant fatigue × direction interactions should be considered inconclusive. In addition, several effects identified by the primary BH procedure did not remain significant under the more conservative BY sensitivity analysis and should be interpreted cautiously. Fifth, the phase-specific quasi-stiffness measure summarizes the net joint moment–angle relationship within a defined movement phase and should not be interpreted as intrinsic joint stiffness or mechanical impedance (Rouse et al., 2013). Because this relationship may be nonlinear, future studies should compare range-based estimates with regression-based or nonlinear approaches. Finally, trials showing obvious slippage or movement discontinuity were discarded and repeated; however, the number of discarded trials was not prospectively recorded, so exact exclusion counts could not be reported. Although trial order was randomized separately within the pre- and post-fatigue sessions, the participant-specific randomization sequences were not retained; therefore, potential trial-order effects could not be examined retrospectively.

## 5 Conclusion

Within the racket-side limb, fatigue induced a more constrained lunge strategy, characterized by a shorter braking phase, reduced multiplanar joint motion, lower recovery-phase anteroposterior impulse, and diminished anteroposterior COM velocity increase. These changes may limit rapid post-lunge repositioning. Compared with forehand lunges, backhand lunges showed greater racket-side impact loading, higher overall, mediolateral, and vertical dynamic postural stability index values, a greater knee extension moment, and greater ankle motion and stability-control demands, whereas forehand lunges showed higher anteroposterior stability index values. No statistically significant fatigue × direction interactions were detected after BH correction; however, given the limited statistical sensitivity for interaction effects, these findings should be considered inconclusive rather than evidence of an absence of interaction. From a practical perspective, coaches may consider monitoring lower-limb movement control and repositioning capacity when evaluating lunge performance under fatigue. Training emphasizing eccentric strength, force production, and neuromuscular control may help preserve braking and recovery mechanics. However, these findings should be interpreted within the context of a controlled laboratory fatigue protocol and do not directly indicate injury risk or match-specific performance outcomes.

## Data Availability

The raw data supporting the conclusions of this article will be made available by the authors, without undue reservation.
